# The association of cemiplimab plus sonidegib for synchronous cutaneous squamous cell carcinoma and basal cell carcinoma of the head and neck: Two case reports

**DOI:** 10.3389/fonc.2023.1111146

**Published:** 2023-02-28

**Authors:** Elena Colombo, Cristina Gurizzan, Arianna Ottini, Francesca Caspani, Cristiana Bergamini, Laura D. Locati, Chiara Marchiselli, Andrea Alberti, Luigi Lorini, Lisa F. Licitra, Paolo Bossi, Carlo Resteghini

**Affiliations:** ^1^ Head and Neck Medical Oncology Unit, Fondazione IRCCS Istituto Nazionale dei Tumori, Milan, Italy; ^2^ Division of Medical Oncology, Department of Medical and Surgical Specialties, Radiological Sciences and Public Health, University of Brescia, ASST Spedali Civili, Brescia, Italy; ^3^ Department of Oncology and Hematology , University of Milan, Milan, Italy; ^4^ Department of Oncology and Hematology , University of Brescia, Brescia, Italy

**Keywords:** cutaneous squamous cell carcinoma, basal cell carcinoma, synchronous carcinomas, immunotherapy, checkpoint inhibitors, hedgehog inhibitors, case report

## Abstract

Basal cell carcinoma (BCC) and cutaneous squamous cell carcinoma (cSCC) are the most frequent cancers in humans, with cumulative ultraviolet radiation exposure, aging, and immunodepression as the main risk factors. In most cases, these malignancies arise in the head and neck area, and they can be treated with locoregional therapies. A minority of cases require systemic therapy. Currently, Sonic Hedgehog inhibitors (i.e., vismodegib and sonidegib) have been approved for advanced BCC, while the PD-1 checkpoint inhibitor cemiplimab has been approved as a first-line treatment for cSCC and as a second-line treatment for BCC. Nevertheless, there is a clinical need for an effective and safe systemic therapies for advanced synchronous (syn) BCC/cSCC not amenable to local treatments. International guidelines do not provide specific recommendations for patients affected by this condition, and no case reports on the full-dose association of these medications have been previously reported. Here, we present the cases of two elderly patients affected by synBCC/cSCC of the head and neck, who received combined therapy with cemiplimab and sonidegib at full dose and standard schedule, achieving remarkable clinical benefit and long-term responses, without major adverse events. The instance of a feasible treatment for patients with advanced synBCC/cSCC will become increasingly frequent with the advancement of life expectancy in the global population, and the synergistic activity of targeted therapies and immunotherapy—administered either in association or sequentially—deserves to be further explored.

## Introduction

**Figure 1:**
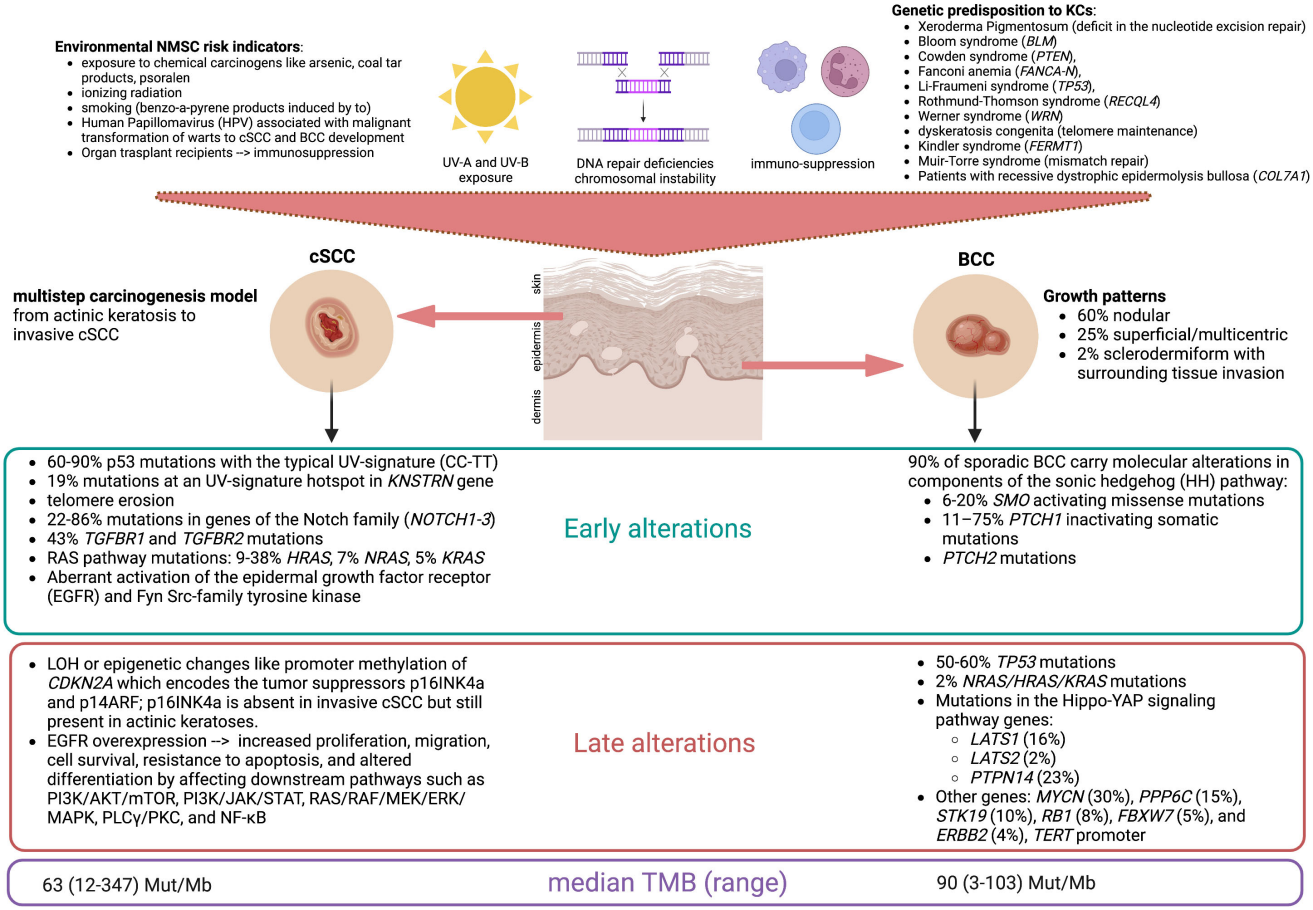
Keratinocytes are the prevalent resident cell types within the epidermis. Common risk factors of keratinocyte cancers include sun exposure, DNA repair deficiencies leading to chromosomal instability, and immunosuppression. Frequently, these factors are intermingled, as DNA photoproducts are almost exclusively repaired by the nucleotide excision repair pathway, and cancerous cells escape from detection in the presence of a defective immune system. This figure reports the main risk factors for the development of cutaneous squamous cell carcinoma (cSCC) and basal cell carcinoma (BCC) with the most frequent molecular alterations found in the early and late developments of these lesions and their relative prevalence (%). The median tumor mutational burden (TMB), expressed in the number of mutations per megabase (Mut/Mb), is correlated with genomic instability and is considered high if >10 Mut/Mb. BCCs harbor a low level of immune cell infiltration, but HHi therapy increases CD8+ and CD4+ T-cell infiltration and sensitivity to immune checkpoint inhibitors. (Figure created by the authors with BioRender).

Non-melanoma skin cancers (NMSCs) are the most common malignancies in humans. Keratinocyte cancers (KCs) as basal squamous cell carcinoma (BCC) and cutaneous squamous cell carcinoma (cSCC) account for 99% of NMSCs, with a BCC-to-SCC prevalence ratio between 1:1 and 10:1, depending on the population, ethnic group, and gender (1). In the general population, BCC has higher incidence rates than cSCC, while the reverse is seen in the population of solid organ transplant recipients (2). Advanced cSCC has higher lethality than BCC, and the overall incidence of cSCC increased by more than 200% between 1990 and 2019 (3). The DNA damage from ultraviolet (UV-A and UV-B) radiation exposure is the major risk factor for both histotypes; BCC is caused by intensive UV exposure in childhood/adolescence, while cSCC is related to cumulative UV exposure over decades (**Figure 1**) (4). Synchronous BCC/cSCC (synBCC/cSCC) is not infrequent: the 3-year cumulative risk of developing a BCC in patients with a prior SCC is 44%, while the risk of developing a cSCC in patients with a prior BCC is approximately 6% (5). In a recent retrospective study on the Australian population, the 5-year cumulative risk of subsequent keratinocyte carcinoma after a first diagnosis of synBCC/cSCC was 51%, with an annualized 5-year incidence rate of 16,634/100,000 person-years at risk (6). Another retrospective study on 969 patients diagnosed in Portugal observed that cases with a history of skin cancer had a risk of 17.0% of developing new skin neoplasms. Moreover, the main risk factors for the development of metachronous lesions were advanced age and detection of synchronous neoplasms at first diagnosis (7). The last GLOBOCAN report showed that, in 2020, more than 63,700 patients succumbed to NMSC-specific death on a global scale. Therefore, the appropriate management of the advanced forms of NMSC is a relevant global health issue (8).

The majority of KCs arise in the head and neck area and can be cured with surgery, cryo-/photodynamic therapy, or radiotherapy (9). Locally advanced BCC (laBCC) and cSCC not amenable to local treatments are aggressive malignancies with low chemosensitivity. Nevertheless, in the last decade, the rise of targeted therapy and immune checkpoint inhibitors has favorably changed the prognosis of patients affected by advanced KCs. Nearly 90% of sporadic BCCs harbor mutations in the genes of the Sonic Hedgehog (HH) pathway, and up to 70% harbor mutations in the *PTCH1* gene on chromosome 9q coding for the patched-1 receptor (10, 11). Hedgehog inhibitors (HHi) are approved by the Food and Drug Administration (FDA) and the European Medical Agency (EMA) for the treatment of unresectable BCCs on the basis of two pivotal studies: the single-arm phase II ERIVANCE BCC with vismodegib given 150 mg orally daily (12) and the phase II BOLT with sonidegib given 200 mg orally daily (dose approved for use) (13). These trials showed an objective response rate (ORR) by central review of 43% and 56%, respectively, although with some differences in the criteria of response evaluation (14).

As both BCC and cSCC harbor a high tumor mutational burden (TMB) due to radiation-induced DNA damage (15, 16), immunotherapy with anti-PD-1 agents has been explored as a potential therapeutic strategy (17–19). The single-arm phase II study EMPOWER-CSCC-1 in a population of patients with cSCC treated with the PD-1 checkpoint inhibitor cemiplimab (350 mg flat dose intravenously every 3 weeks) showed an ORR of 44% (95% CI 32–55), leading to the FDA and EMA approval of first-line treatment for locally advanced/metastatic cSCC (20). In February 2021, the FDA approved cemiplimab also for the treatment of laBCC in patients who progressed on HHi or were intolerant to prior HHi therapy, on the basis of a non-randomized phase II trial reporting an ORR of 31% (95% CI 21–42), with 6% of complete responses and 25% of partial responses; serious treatment-related adverse events (AEs) occurred in 35% of the study population (21). Recently, a phase II basket study (NCT03012581) included 32 advanced BCC patients to evaluate the efficacy and safety of the PD-1 inhibitor nivolumab after the failure of HHi. Disease control was achieved in terms of complete responses (12.5%), partial responses (18.8%), and stable diseases (43.8%) (22). Despite the high TMB, BCC has a low immune infiltration, but HHi therapy increases CD8+ and CD4+ T-cell infiltration, suggesting a synergistic effect of the combination/sequence of treatment with HHi and checkpoint inhibitors on the management of laBCC (23).

Currently, the international guidelines do not provide specific recommendations for the first-line therapy of patients with advanced synBCC/cSCC. Therefore, clinicians face complex decisions for the treatment of these cases in real-world practice. A previous proof-of-principle non-randomized trial on 16 patients with laBCCs tested anti-PD-1 pembrolizumab with (*n* = 7) or without (*n* = 9) vismodegib, reporting an overall response rate (ORR) of 29% for the anti-PD-1/HHi doublet and 44% for anti-PD-1 monotherapy (24). Investigators concluded that pembrolizumab is active against BCCs and that the response of the anti-PD-1/HHi group was not superior to the anti-PD-1 monotherapy group; however, no specific information about the safety of anti-PD-1/HHi combination was provided. Here, we report two cases of synchronous laBCC and cSCC of the head and neck area treated with cemiplimab and sonidegib in two different tertiary referral centers. Both patients gave their written permission to use information—including photographs—about their clinical history for the purposes of this paper. All the reported AEs are graded according to the Common Terminology Criteria for Adverse Events (CTCAE) version 5.0 (25). The staging was given according to the clinical TNM Staging System of the cSCC and BCC of the head and neck (AJCC/UICC 8th Edition).

## Case 1: Cemiplimab plus sonidegib

**Figure 2:**
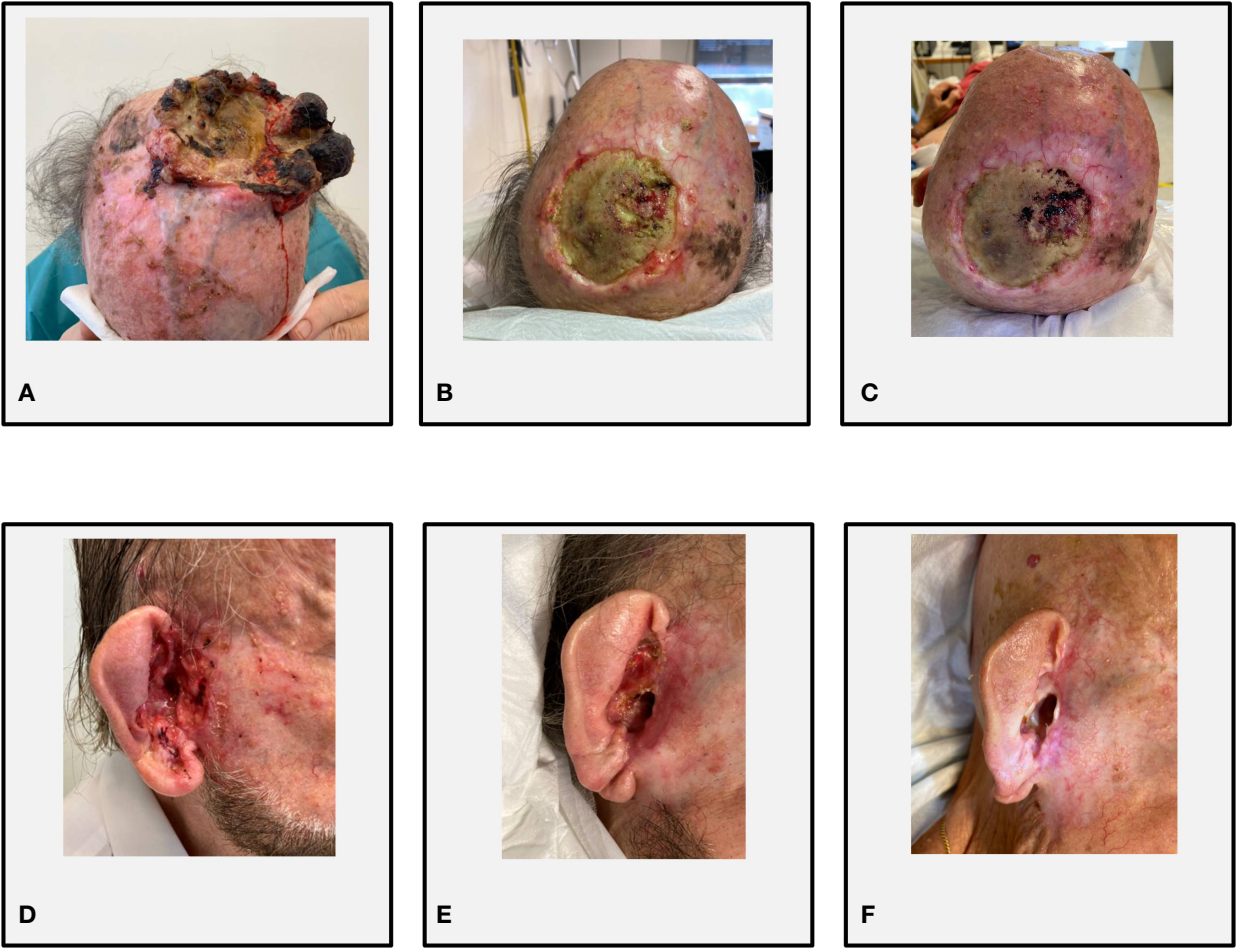
Case 1. **(A)** Bulky recurrence on the vertex of cSCC at cycle 1 (C1) of cemiplimab; **(B)** partial response at C23 of cemiplimab and C4 of sonidegib; **(C)** stable disease at C30 of cemiplimab and C10 of sonidegib. **(D)** Infiltrative BCC extended to the right external auditory meatus at C18 of cemiplimab and C1 of sonidegib; **(E)** partial response at C23 of cemiplimab and C4 of sonidegib; **(F)** complete response of BCC at C30 of cemiplimab and C10 of sonidegib.

A 76-year-old Caucasian man with a history of multiple excisions of localized KCs was referred for a bulky recurrence of cSCC with sarcomatoid features of the vertex. The lesion had been treated 20 months before with R1 surgery (positive lateral margins) followed by three courses of palliative hemostatic radiotherapy, the last delivered 1 month before the first visit to our institution. The patient presented with a large and painful vegetating mass of the vertex characterized by a fibrinous central ulcerated–hemorrhagic area and peripheral necrosis (**Figure 2A**). The baseline skin examination revealed multiple BCCs, mostly localized in the head and neck region. The patient had a good performance status (ECOG PS 1) and did not present significant comorbidities; he did not report a family history of malignant neoplasms of the skin. A whole-body computer tomography scan excluded distant metastases (cTNM cT3N0M0). On October 2019, the patient started cemiplimab 350 mg intravenously every 3 weeks, achieving a dimensional reduction of the cSCC on the vertex (**Figures 2B, C**) and numerical reduction of the multiple diffuse BCCs, without reporting immune-related adverse events (irAEs). After 6 months from the start of immunotherapy, the patient reported rapid onset of a painful, hemorrhagic lesion in the right auricular area. At clinical examination, the ulcerated lesion was infiltrating the external auditory meatus, and a biopsy confirmed the clinical suspect of BCC (cT3) (**Figure 2D**). A multidisciplinary evaluation deemed the lesion not amenable to radical surgery or radiotherapy. In order to maximize the symptomatic control and maintain the clinical benefit of the cSCC, sonidegib at standard dose and schedule was started in November 2020, while continuing cemiplimab administration at the usual dose. A prompt clinical response on the laBCC was observed (**Figures 2E, F**), while the cSCC at the vertex maintained clinical and radiological partial response. On the last follow-up visit, the patient had completed 31 cycles of cemiplimab and 10 cycles of sonidegib. The combined treatment was well tolerated, with mild HHi-related AEs (myalgia G1, alopecia G1) and no evidence of irAEs. In August 2021, the patient succumbed to the consequences of sepsis, deemed unrelated to the oncological treatment.

## Case 2: Sonidegib followed by cemiplimab

**Figure 3:**
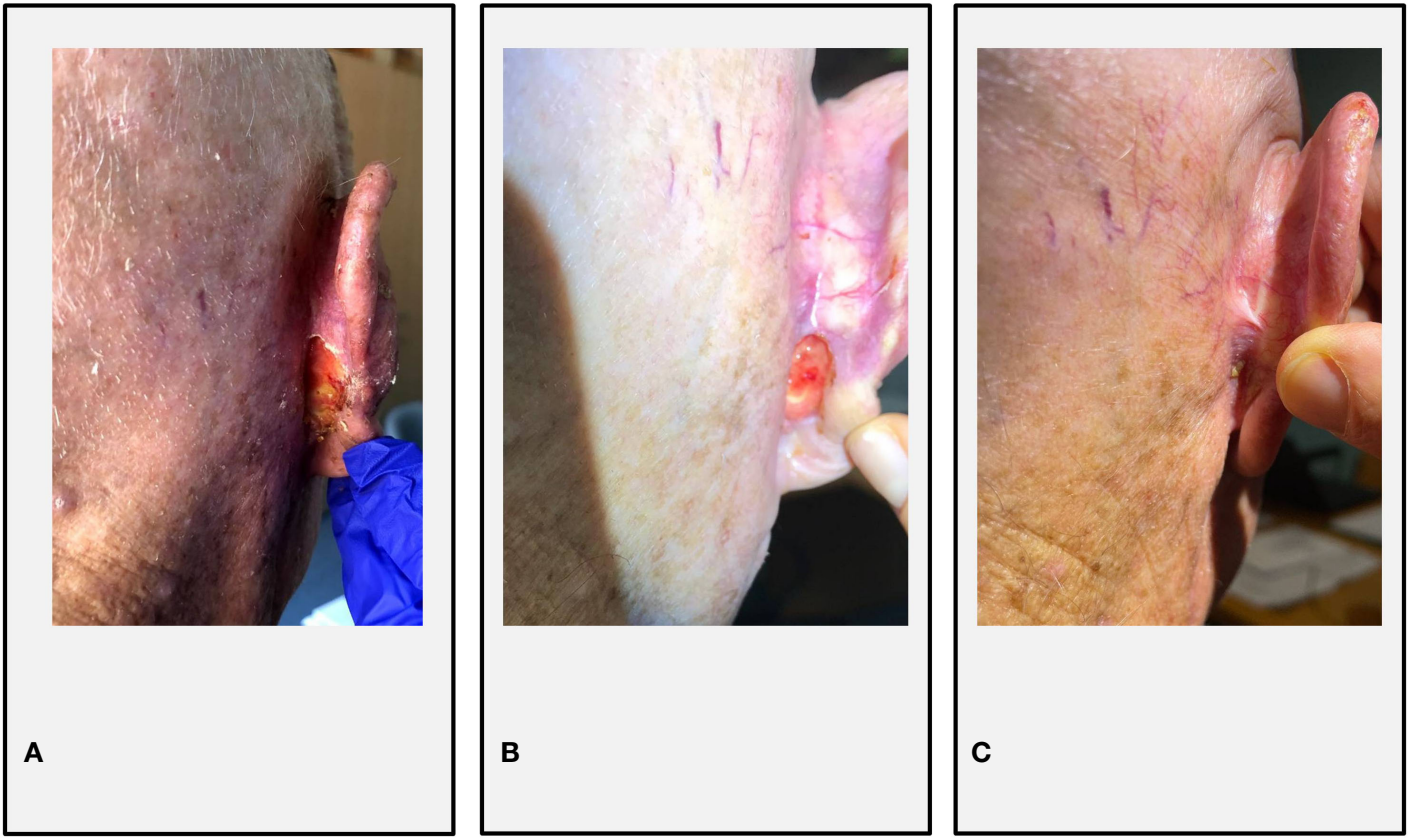
Case 2. **(A)** Retroauricular BCC infiltrating the right auricular cartilage at C1 of sonidegib; **(B)** partial response at C10 of sonidegib; **(C)** complete response at C10 of cemiplimab. A computer tomography scan of the neck reported a complete response of the cSCC nodal relapse at C7 of cemiplimab.

An 83-year-old Caucasian man with a history of multiple excisions of head and neck cSCCs and BCCs was evaluated for the onset of 3-cm right fronto-temporal recurrence of cSCC (cT2) and a painful right retroauricular BCC infiltrating the auricular cartilage (cT3) (**Figure 3A**). The patient had a good performance status (ECOG PS 1) and did not present significant comorbidities; he did not report a family history of malignant neoplasms of the skin. After a multidisciplinary evaluation, in April 2020, the patient underwent a multimodal therapeutic approach to the lesions. Surgical removal of the right fronto-temporal cSCC was performed (R0, with perineural invasion), followed by adjuvant radiotherapy (total dose 66 Gy in 33 fractions); meanwhile, he started systemic treatment with sonidegib. HHi therapy was well tolerated, except for mild dysgeusia and asthenia (G1). After 2 months of sonidegib therapy, a significant clinical reduction of the retroauricular BCC was achieved (**Figure 3B**). In December 2020, a right retroangulomandibular adenopathy occurred, histologically diagnosed as cSCC metastasis. Since neither surgery nor radiotherapy was deemed feasible, cemiplimab at standard dose was started. Sonidegib was discontinued after 10 cycles, considering the good radiological response obtained at the retroauricular level and the HHi-related toxicities reported (muscle spasms G2, dysgeusia G2, and asthenia G2). The patient experienced a mild infusion reaction at the first cycle (lower back pain and flushing). At the last follow-up on 22 November 2022, the patient completed 27 cycles of cemiplimab, obtaining a complete response with clinical benefit (**Figure 3C**) without significant AEs from the sequential HHi/anti-PD-1 therapy.

## Discussion

**Figure 4:**
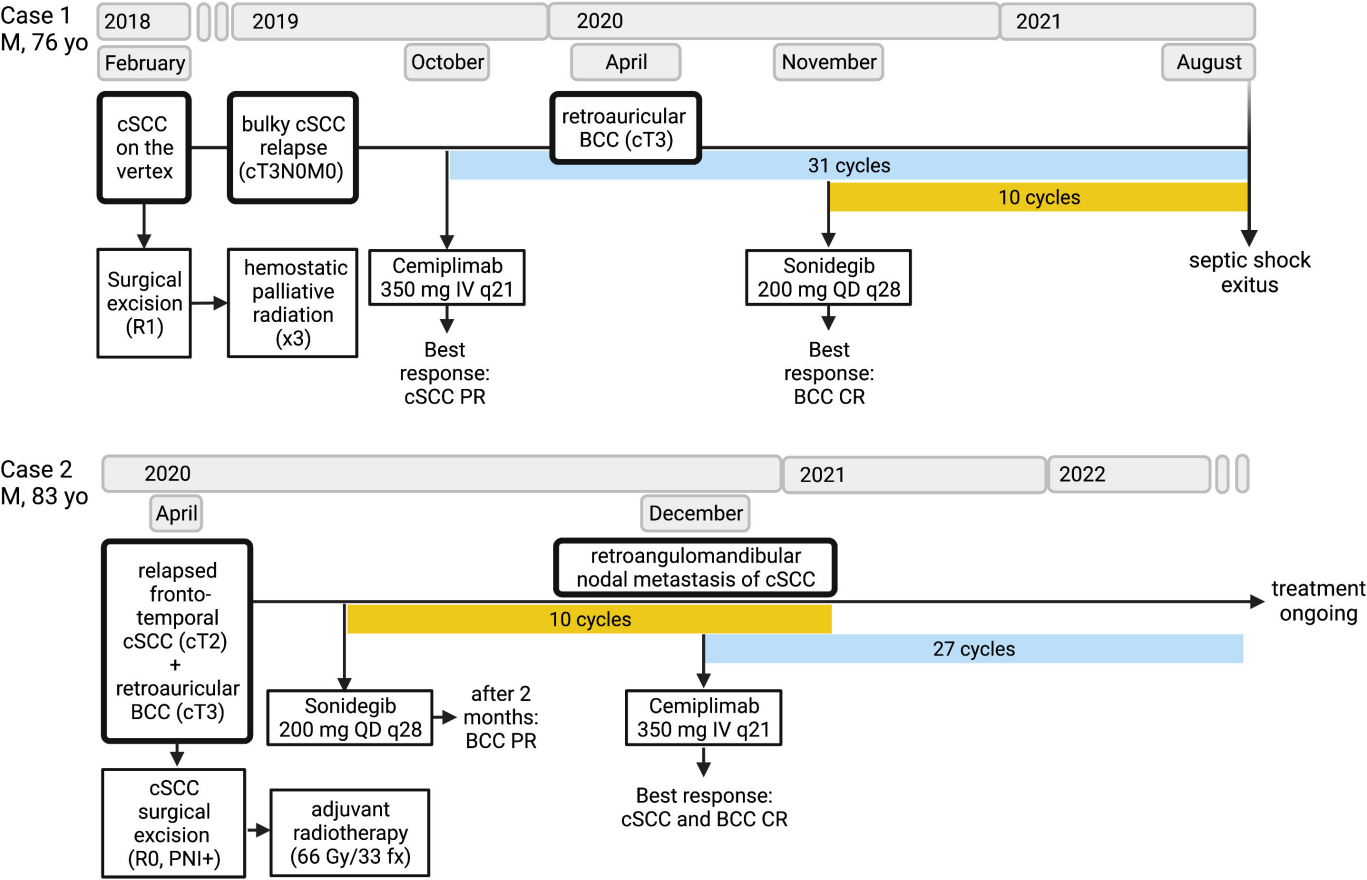
Timeline showing the multimodal treatments received and the responses achieved by each patient since surgery to systemic therapy. The clinical staging of cutaneous squamous cell carcinoma (cSCC) and basal cell carcinoma (BCC) is based on the TNM AJCC/UICC 8th Edition criteria. For resected lesions, surgical margin status (R1, microscopically positive; R0, negative) and positive perineural invasion (PNI+) are reported. Adjuvant radiotherapy treatment is quantified in Gray (Gy) per fraction (fx). Blue blocks correspond to cemiplimab immunotherapy given intravenously (IV) at the flat dose of 350 mg once every 21 days (q21); yellow blocks correspond to sonidegib targeted therapy given orally every day (QD) in cycles of 28 days (q28). Responses are estimated according to the RECIST v.1.1 criteria (PR, partial response; CR, complete response). (Figure created with BioRender).

To the best of our knowledge, these are the first clinical reports on the concomitant and sequential full-dose administration of sonidegib and cemiplimab in patients with inoperable synchronous laBCC/cSCC. In our cases, the HHi/anti-PD-1 association proved to be an effective and well-tolerated strategy. In Case 1, the preauricular laBCC was resistant to immunotherapy, and the association of sonidegib with cemiplimab enabled achieveing a complete response of the laBCC, while maintaining the benefit from anti-PD-1 on the cSCC. In Case 2, sonidegib was started to treat a retroauricular laBCC, achieving a partial response; then, cemiplimab was started for a nodal relapse of cSCC, obtaining a complete response of both lesions. These two different therapeutic approaches to synchronous laBCC/SCC, imbricating the PD-1 inhibitor cemiplimab with HHi in concurrent and sequential ways, were both well tolerated despite the age of the patients, who managed to continue the systemic treatments for almost 22 months in Case 1 and at least 30 months in Case 2 (**Figure 4**).

Over the last 5 years, the landscape of treatments for advanced KCs has profoundly changed, and HHi/anti-PD-1 combination therapy is currently under evaluation. The Sonic Hedgehog pathway promotes tumor-associated macrophage polarization (26) and induction of PD-L1 expression (27). Therefore, HHi may promote an adaptive immune response in BCC, fostering a synergistic effect with anti-PD-1 checkpoint inhibitors (28).

Ongoing clinical trials are evaluating the combination of anti-PD-1 plus HHi in laBCC. The design of a phase II open-label non-randomized clinical trial with cemiplimab and sonidegib administered in a pulsed schedule was presented at the ESMO Congress 2021 (NCT04679480): the study planned to include 20 patients with advanced BCC receiving sonidegib in a run-in phase, followed by combination therapy of cemiplimab at standard dose plus sonidegib in a 2-week cycle every 4 weeks (pulsed therapy: 2 weeks on, 2 weeks off) to evaluate primarily the best response at any time between treatment start and 26 weeks after the initiation of the treatment, and secondarily the tumor response at 26 weeks, changes in histology/immunogenicity of the tumor, and treatment safety (29). In addition, a phase I study with pembrolizumab and sonidegib for solid malignancies is currently ongoing, including patients with stage IV cSCC of the head and neck as well (NCT04007744).

Even though laBCC and unresectable cSCC have comparable risk factors and immunologic characteristics, they are two entities with different pathogenesis, molecular alterations, clinical presentation, and evolution (30, 31). Despite a common sensitivity to PD-1 inhibitors, the spectrum of responses is heterogeneous and reflects the microenvironment composition. Therefore, instead of a one-size-fits-all approach with immunotherapy, our report highlights the opportunity of considering the two components of advanced synchronous BCC/cSCC as distinct malignancies that may require—at least in certain cases—a systemic treatment with both HHi and PD-1 inhibitors in order to achieve symptomatic control and durable objective responses.

## Data availability statement

The raw data supporting the conclusions of this article will be made available by the authors, without undue reservation.

## Ethics statement

Written informed consent was obtained from the individual(s) for the publication of any potentially identifiable images or data included in this article.

## Author contributions

EC, CG, AO, FC, CB, LDL, CM, AA, LL, LFL, PB, and CR participated in the concept design of the manuscript. EC, CG, PB, and CR drafted the introduction and discussion sections. EC designed **Figures 1** and **4**. AO, FC, and CB retrieved the clinical information and pictures of Case 1. CG, AA, and LL retrieved the clinical information and pictures of Case 2. LDL and LFL provided critical feedback on the manuscript content and figures. All authors contributed to the revision of the final manuscript and approved the submitted version.
